# The biomechanical effects of allograft wedges used for large corrections during medial opening wedge high tibial osteotomy

**DOI:** 10.1371/journal.pone.0216660

**Published:** 2019-05-10

**Authors:** James Belsey, Arnaud Diffo Kaze, Simon Jobson, James Faulkner, Stefan Maas, Raghbir Khakha, Dietrich Pape, Adrian J. Wilson

**Affiliations:** 1 Department of Sport, Exercise & Health, University of Winchester, Winchester, Hampshire, England; 2 Basingstoke and North Hampshire Hospital, Basingstoke, Hampshire, England; 3 University of Luxembourg, Faculty of Science, Technology and Communication, Luxembourg, Luxembourg; 4 Department of Orthopaedic Surgery, Centre Hospitalier de Luxembourg, Luxembourg, Luxembourg; 5 The Hampshire Clinic, Basing Road, Old Basing, Basingstoke, Hampshire, England; University of Zaragoza, SPAIN

## Abstract

The inclusion of an allograft wedge during medial opening wedge high tibial osteotomy has been shown to lead to satisfactory time-to-union in larger corrections (>10°). Such large corrections are associated with greater incidences of intraoperative hinge fracture and reduced construct stability. The purpose of this study was to investigate the biomechanical stability that an allograft wedge brings to an osteotomy. Ten medium-size fourth generation artificial sawbone tibiae underwent 12 mm biplanar medial opening wedge high tibial osteotomy with a standard Tomofix plate. Five tibiae had an allograft wedge inserted into the osteotomy gap prior to plate fixation (allograft group). The gap in the remaining tibiae was left unfilled (control group). Each group underwent static compression testing and cyclical fatigue testing until failure of the osteotomy. Peak force, valgus malrotation, number of cycles, displacement and stiffness around the tibial head were analysed. Intraoperative hinge fractures occurred in all specimens. Under static compression, the allograft group withstood higher peak forces (6.01 kN) compared with the control group (5.12 kN). Valgus malrotation was lower, and stiffness was higher, in the allograft group. During cyclical fatigue testing, results within the allograft group were more consistent than within the control group. This may indicate more predictable results in large osteotomies with an allograft. Tibial osteotomies with allograft wedges appear beneficial for larger corrections, and in cases of intraoperative hinge fracture, due to the added construct stability they provide, and the consistency of results compared with tibial osteotomies without a graft.

## Introduction

Medial opening wedge high tibial osteotomy (MOWHTO) is a technique that has gained popularity in comparison to other variations of tibial osteotomy for the treatment of patients with medial osteoarthritis of the knee [1]. When compared to the alternative option of a lateral closing wedge high tibial osteotomy, the MOWHTO is a technically simpler procedure [2, 3] and makes subsequent conversion to total knee arthroplasty easier [2, 4].

Internal plate fixators are often used during MOWHTO and there are many different types of implant on the market. However, it is the Tomofix (Synthes GmbH, Oberdorf, Switzerland) plate that is considered the gold standard, and that has been shown to possess biomechanical properties that promote rapid bone healing [5]. Positive radiological outcomes with the Tomofix plate have been found with both smaller and larger correction angles [6, 7].

Large correction angles of >10° during MOWHTO are associated with higher cases of lateral cortex fractures, either intra-operatively or post-operatively [8, 9]. In turn, such fractures lead to greater instability of the overall construct [1, 3, 10, 11], which can negatively influence certain clinical outcomes such as correction accuracy and time-to-union [3, 10–13].

Studies have shown a negative correlation between the size of an osteotomy gap and time-to-union [1, 14]. However, the addition of an allograft wedge into the osteotomy gap seems to facilitate time-to-union in larger corrections to a satisfactory degree, comparable to smaller osteotomies [14–16]. Many of these findings have also been shown in a recent systematic review investigating the role of bone graft materials in MOWHTO [17]. The authors concluded that there is little evidence regarding the maximum size that an osteotomy gap can be without the need for graft materials. It also suggested that osteotomies with an opening of less than 10 mm should be performed without a graft, except in certain instances that have a high complication risk. The systematic review does not offer a conclusion regarding the use of graft materials in MOWHTO greater than 10 mm, which suggests that this is an area in need of further investigation.

Despite clinically showing satisfactory results, the use of allograft wedges during MOWHTO has never been biomechanically investigated to determine whether they influence the stability of the construct. Such an investigation would be relevant to larger corrections during MOWHTO due to the greater associated risks of lateral cortex fractures and instability.

The purpose of the present study was to investigate the static and fatigue strength of MOWHTO, with a large correction angle, with and without an allograft wedge. It was hypothesised that osteotomies with an allograft wedge would exhibit higher static and fatigue strength than those where no material is inserted into the osteotomy gap.

## Materials and methods

Ten medium-size fourth generation analogue composite tibiae (Sawbones, Pacific Research Laboratories, Inc., Vashon Island, Washington, USA) were used for testing in the present study. Studies into these artificial tibiae have shown them to possess similar biomechanical properties to human bone, and to have much lower inter-specimen variability [18, 19], making them appropriate for research.

### Specimen preparation

**Fig 1:**
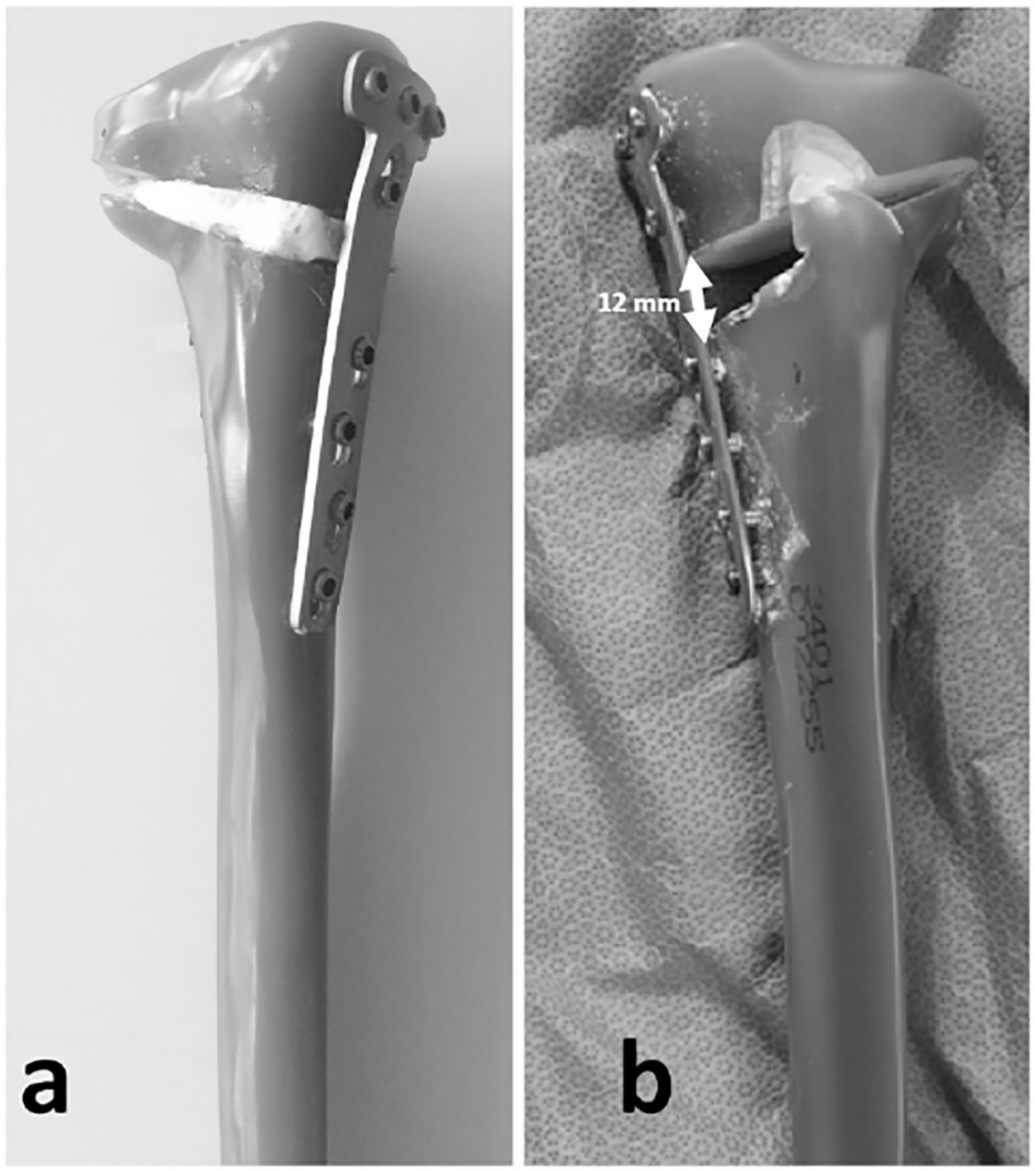
Example specimens from each group. (A) Allograft Group; (B) Control Group.

**Fig 2:**
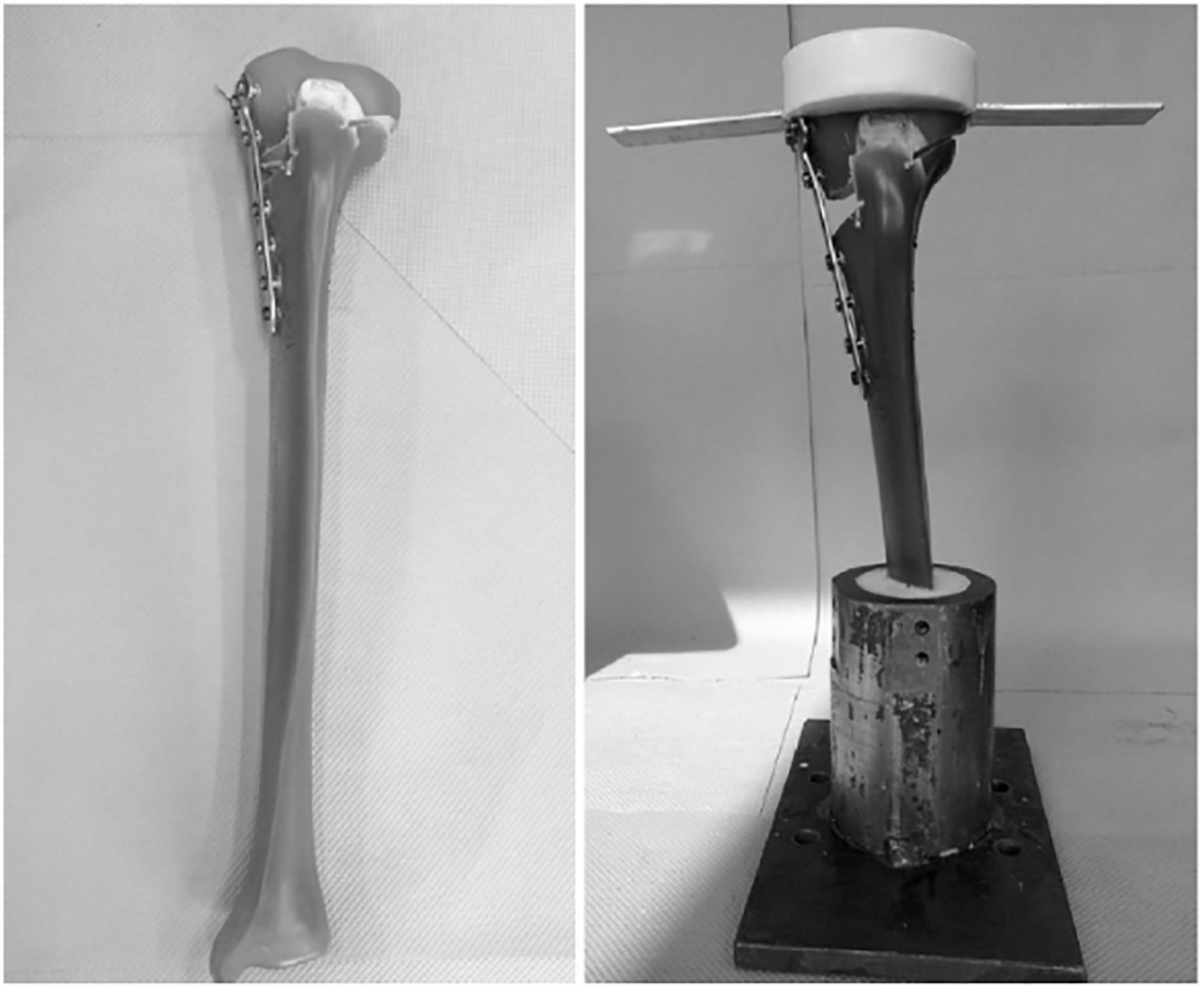
A specimen that has undergone MOWHTO (left) is then prepared for testing (right).

A 12 mm biplanar MOWHTO was performed on each specimen by an experienced orthopaedic surgeon and fixed with a standard Tomofix plate. The osteotomy was performed such that the inclination angle of the tibial plateau was horizontal in the frontal and sagittal planes. In five specimens, a 12 mm HTO wedge allograft (RTI Surgical Inc., Alachua, USA), sourced from the proximal tibia of a donor, was inserted prior to plate fixation (Allograft Group) (Fig 1A) and held in place using an ethyl cyanoacrylate glue. In the remaining five tibiae, the osteotomy gap was left unfilled (Control Group) (Fig 1B). Each specimen was then prepared for testing (Fig 2) following the protocols described by Maas *et al*. (2013) and Diffo Kaze *et al*. (2015).

### Static test protocol

**Fig 3:**
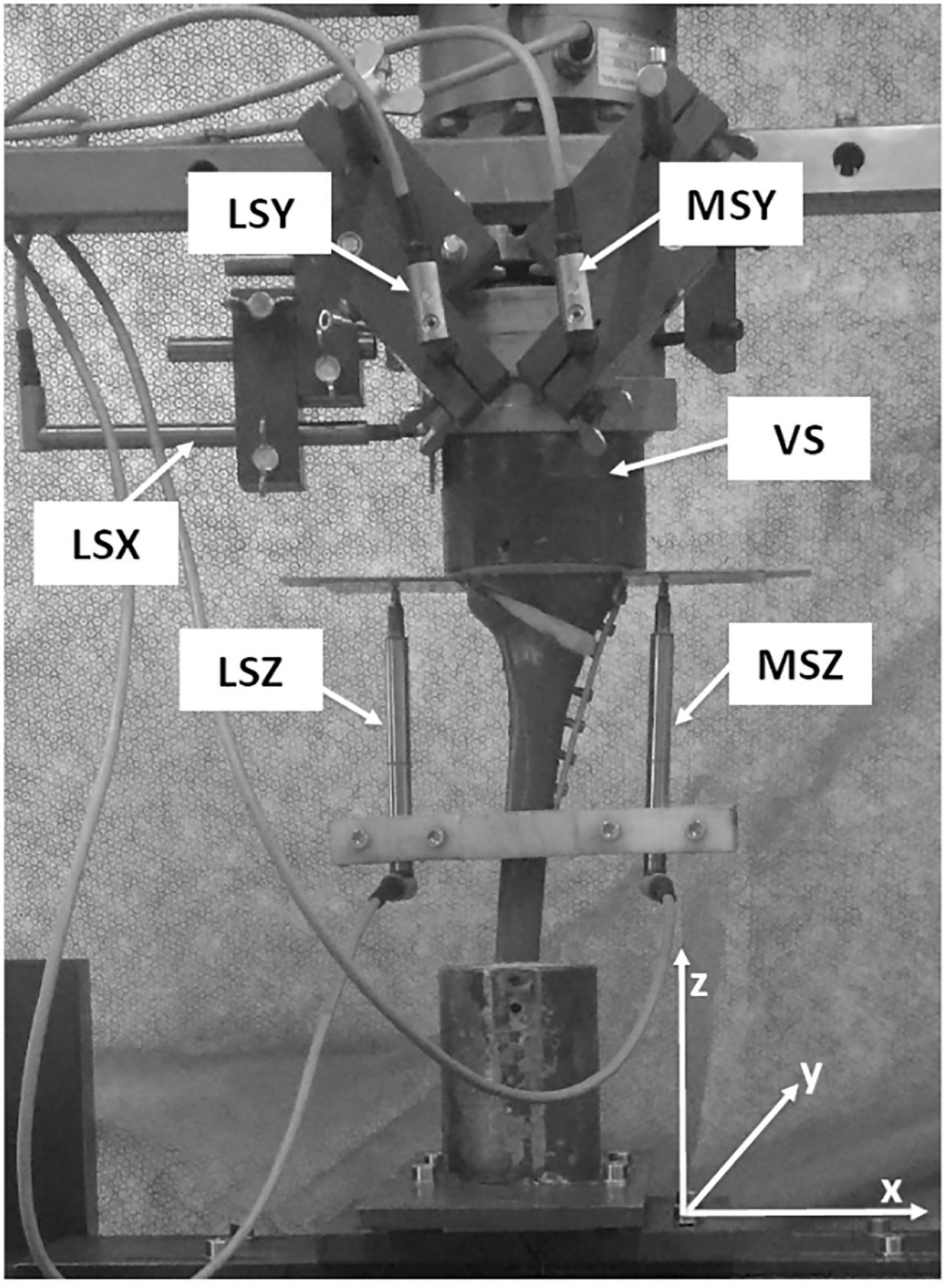
Positioning of displacement sensors around the tibial head (posteromedial view). Abbreviations: LSX = Lateral Sensor X-Axis; LSY = Lateral Sensor Y-Axis; LSZ = Lateral Sensor Z-Axis; MSY = Medial Sensor Y-Axis; MSZ = Medial Sensor Z-Axis; VS = Vertical Sensor.

Following the protocol described by Diffo Kaze *et al*. (2015), two specimens from each group underwent static testing. Each specimen was loaded onto a 10kN hydraulic piston (INSTRON, Darmstadt, Germany), which applied an axial load to the tibial head through a freely moveable support, which contained three metal balls that allowed the support freedom of movement in the transverse plane. The distal end of the specimen was screwed down to the piston, preventing the deep cylindrical mould from moving in the transverse plane. Six displacement sensors were used to measure the level of deformation at various positions around the tibial head. With reference to the transverse plane, five of the sensors were positioned as follows (Fig 3): lateral to the tibial head in the x-axis (LSX); medially and laterally to the tibial head in the y-axis (MSY and LSY, respectively); and medially and laterally to the tibial head in the z-axis (MSZ and LSZ, respectively). The sixth sensor (VS) was contained within the test machine itself and measured the vertical displacement of the hydraulic piston.

The piston then applied static compression to the specimens under displacement-controlled conditions at a rate of 0.1 mm·s^-1^ until failure of the osteotomy. Failure was defined as being the point at which the lateral cortex of the tibial head collapsed. This was something that could be seen and heard, as well as measured by a sudden drop in the force being applied by the piston.

### Fatigue strength test protocol

Following the protocol of a previous study [5], the remaining three specimens from each group underwent fatigue strength testing. Each specimen was loaded onto the piston, and displacement sensors attached, as described above.

**Fig 4:**
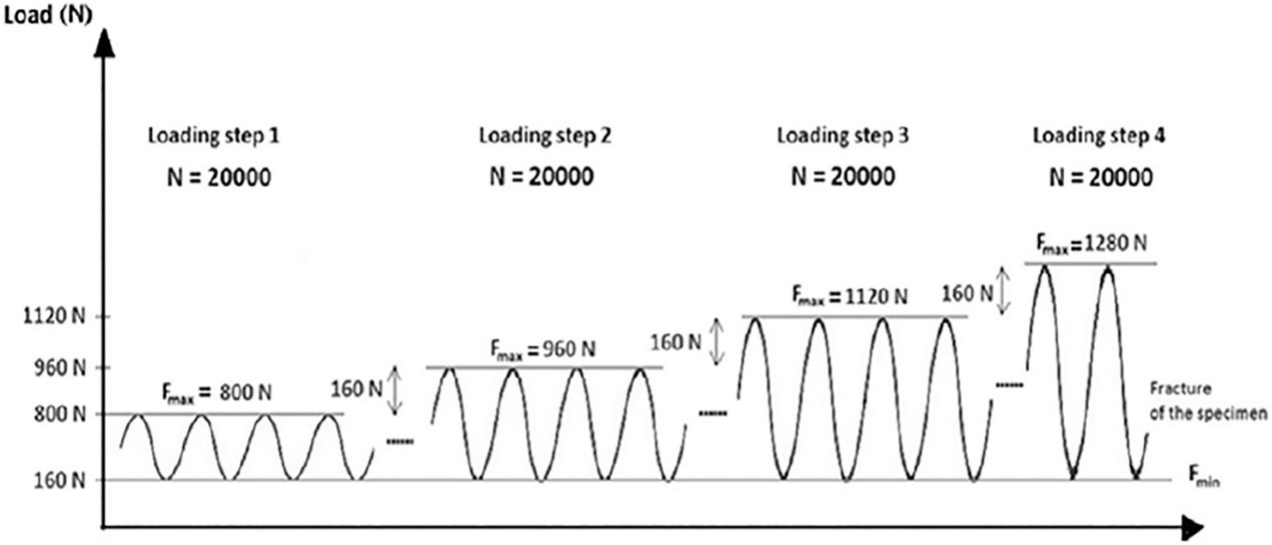
Applied vertical sinusoidal force step loading [21]. Loading frequency remained constant at 5 Hz and the upper force limit increased 0.16kN stepwise every 20,000 cycles until failure.

Sinusoidal loading at a frequency of 5 Hz was then applied by the piston to each specimen. Compression was increased stepwise until the point of failure at the lateral cortex of the tibial head (Fig 4). The lower compressive force limit remained constant at 0.16kN throughout each load step. The upper compressive force limit for the first step was 0.8kN, which was then increased at a constant rate of 0.16kN after every 20,000 cycles (one load step), if the specimen remained intact.

### Analysis

Due to the small sample size in the present study, statistical analysis was not performed on the data and only the means have been reported, as has been done previously [5]. Peak force (kN) and displacement (mm) of each sensor at the point of specimen failure was recorded. Displacements were measured as either positive or negative values, which indicated the direction of the displacement as well as the distance travelled.

**Fig 5:**
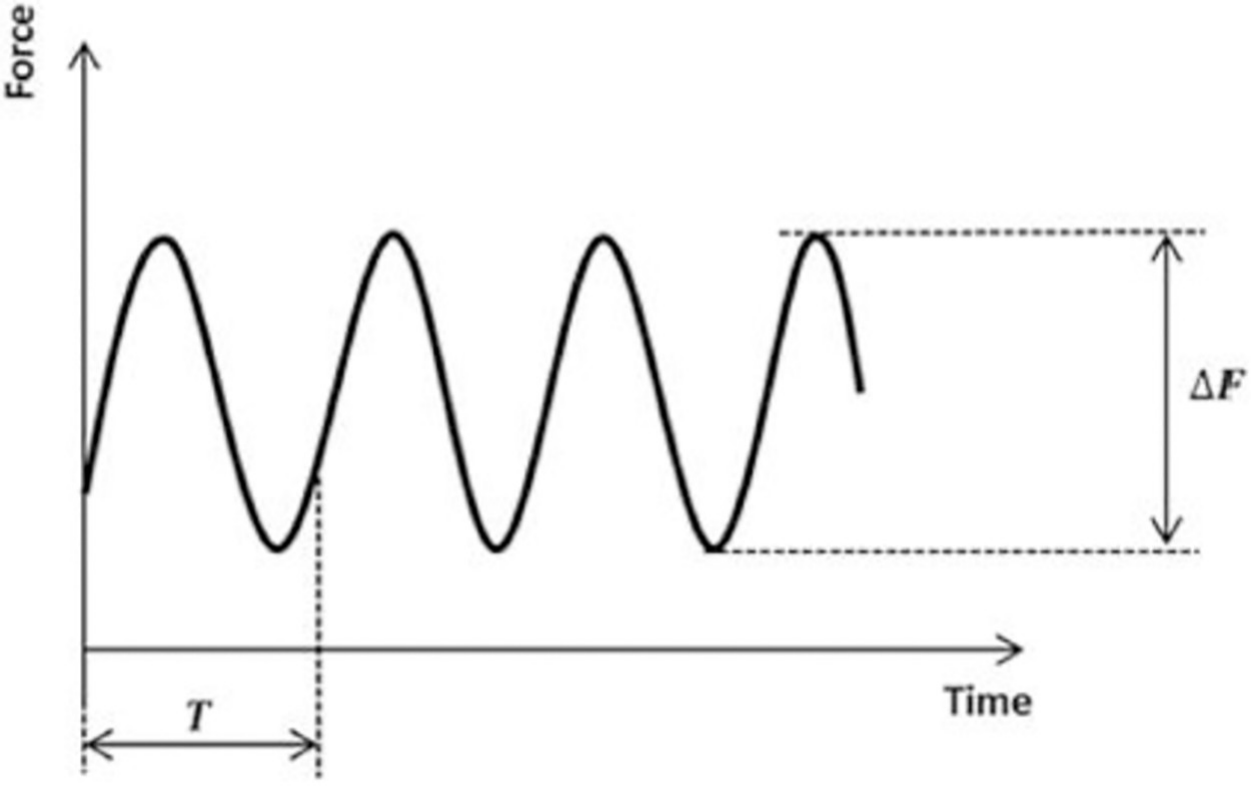
Definition of ΔF and ΔX for the calculation of specimen dynamic stiffness during fatigue strength testing [21]. This is achieved by calculating the ratio of the peak-to-peak forces (ΔF) and the corresponding peak-to-peak displacements (ΔX) within the same time period.

Dynamic stiffness of the specimen throughout each fatigue strength test was calculated using the ratio of the peak-to-peak force and peak-to-peak displacement from the same period of time at each sensor position around the tibial head. For the static tests, specimen stiffness at each position was determined by calculating the ratio of the peak forces (ΔF) and displacements (ΔX) at the point of failure (Fig 5) [5, 20, 21]. For these specimens, any negative displacement values were multiplied by -1, prior to calculation of stiffness, in order to make them positive. This meant that only positive values were used, since the direction of the displacement is irrelevant for this calculation.

Additionally, valgus malrotation of the tibial head was calculated for all specimens that underwent static testing. This was done by using the following formula from Diffo Kaze *et al*. (2015):

$$\alpha =\frac{\left|d_{L}-d_{M}\right|}{D}$$

Where “*α*” is the valgus malrotation (rad), “*d*_*L*_” is LSZ displacement (mm), “*d*_*M*_” is MSZ displacement (mm), and “*D*” is the distance between the two sensor positions. The value “α” was then converted from radians to degrees by multiplying “α” by 180°/3.14 rad.

### Specimen allocation

Due to hardware limitation, the specimens were initially grouped in a way similar to research previously described by Diffo Kaze *et al*. (2015) i.e. 2 specimens for each group for static testing and 3 per group for fatigue strength testing.

### Statistical analysis

The number of specimens was limited due to financial reasons; therefore, no power analysis was performed. A t-test for two independent samples was used to compare the control group and the allograft group using Microsoft Excel 2010 software (Microsoft Corporation, Redmond, Washington, USA). All statistical tests were performed two tailed. Statistical significance was considered at p<0.05.

### Ethics

Ethical approval for this study was granted by the University of Winchester Faculty of Business, Law & Sport ethics panel.

## Results

**Fig 6:**
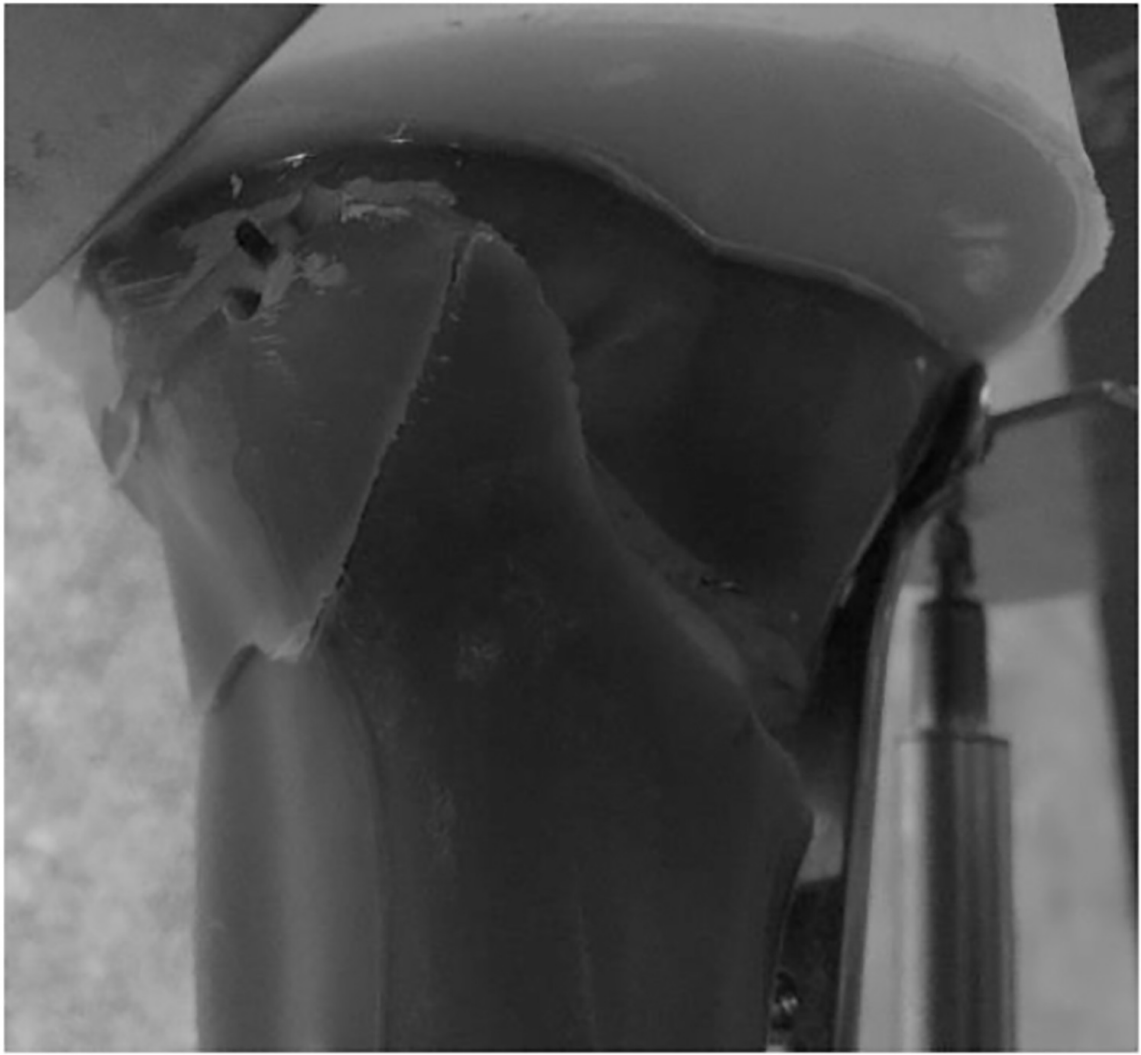
Example of lateral cortex fracture indicating failure of the construct.

All specimens exhibited a lateral hinge fracture intraoperatively. A system malfunction during a fatigue test destroyed one tibia (specimen 1) from the Allograft Group, meaning that the data from this specimen could not be used in the analysis. In all tested specimens, except for one tibia (specimen 3) in the Allograft Group undergoing fatigue strength testing, construct failure occurred due to further fracture of the lateral cortex of the tibial head (Fig 6). Testing of specimen 3 from the Allograft Group was halted due to excessive valgus malrotations causing the lower safety limits to be tripped on the test machine. This was considered a specimen failure, and the data were included in the analysis. Since the specimen was not visibly damaged (other than the intra-operative hinge fracture), it also underwent static compression to failure.

The following analyses were based on: 2 specimens with an allograft, and 3 specimens with no graft, undergoing fatigue strength testing; and 3 specimens with an allograft, and 2 specimens with no graft, undergoing static strength testing.

### Static compression tests

Cracking was observed in one specimen from each group prior to the final failure of the specimen. This cracking was first observed at a force of 3.78 kN in the control group, and at 3.12 kN in the allograft group. Table 1 shows the mean peak force (kN) ± standard deviation (SD) and time (s) at the point of failure for each group. The allograft group withstood higher loads until construct failure than the control group.

**Table 1:** Mean force at time of failure in each group.

Group	Mean Force (kN) at Time of Fracture	Time (s) at Point of Fracture
Control	5.12	40.36
Allograft	6.01	44.54

**Fig 7:**
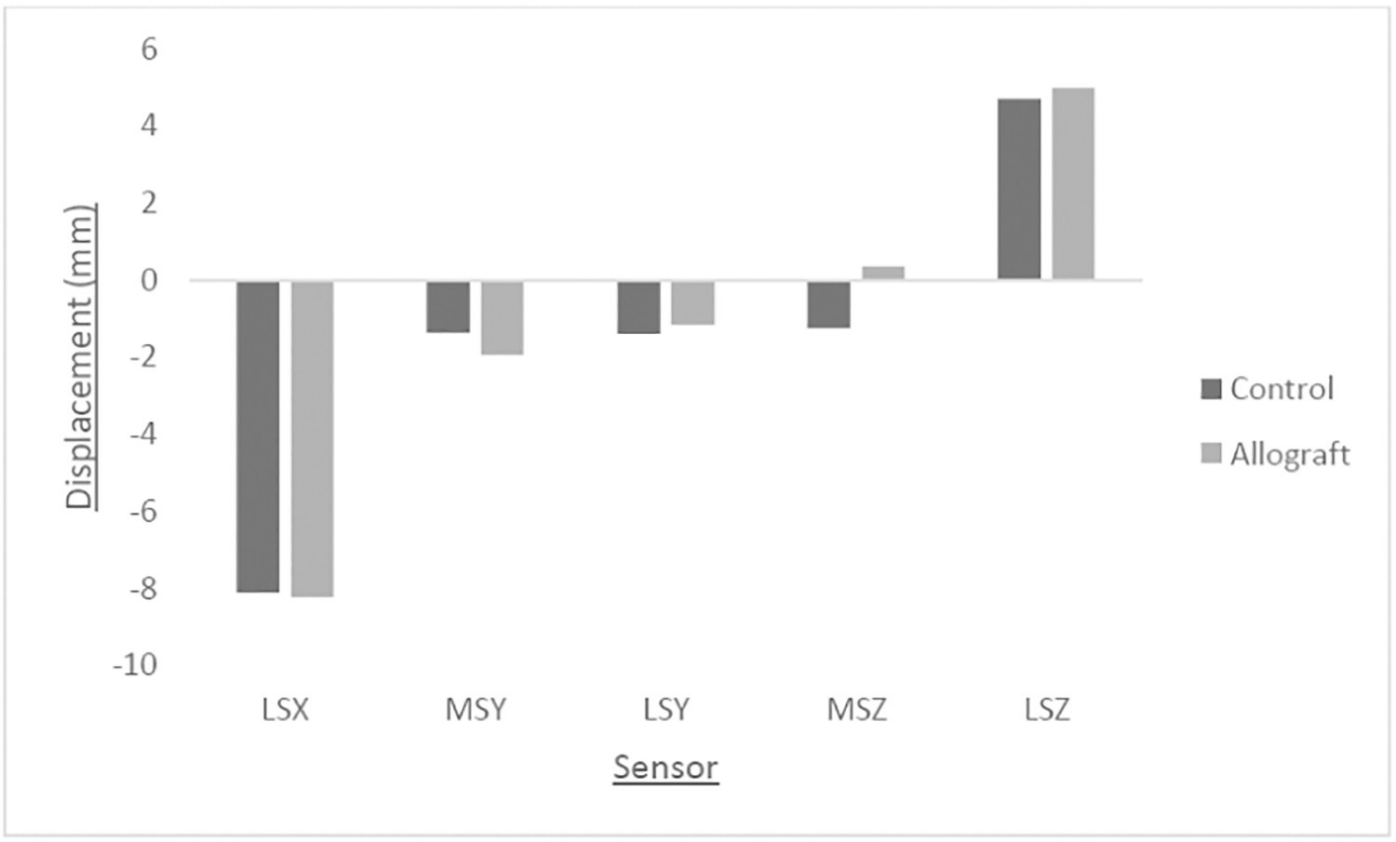
Mean displacement (mm) at each sensor position around the tibial head at specimen failure. Negative values at LSX indicate lateromedial movement. Negative values at MSY and LSY indicate posteroanterior movement. Negative and positive values at MSZ and LSZ indicate vertical upward and downward movements, respectively.

Fig 7 shows the mean displacements at the point of failure at each sensor position around the tibial head. The largest absolute displacement in both groups was seen at position LSX. This is due to the fact that the tibia head could move freely in the transverse plane. The negative LSX values indicate movement in a lateromedial direction. Values in both groups at position MSY and LSY were negative, indicating a posteroanterior movement of the tibial head. Since the values between these two sensor positions were not similar within groups, a slight axial rotation of the tibial head is also indicated. The allograft group showed a positive displacement at position MSZ, whereas the control group showed a negative displacement, indicating vertical downward and upward movements, respectively. LSZ displacement values were positive for both groups, indicating an overall vertical downward displacement. The difference in values within groups at position LSZ also indicate valgus malrotation of the tibial head. Since the control group displayed a negative displacement at MSZ but a positive displacement at LSZ, and the allograft displayed positive values at both of these positions, larger valgus malrotation of the tibial head is indicated in the control group. Valgus malrotation of the tibial head was calculated and was found to be lower in the allograft group (2.22°) than in the control group (2.85°).

**Fig 8:**
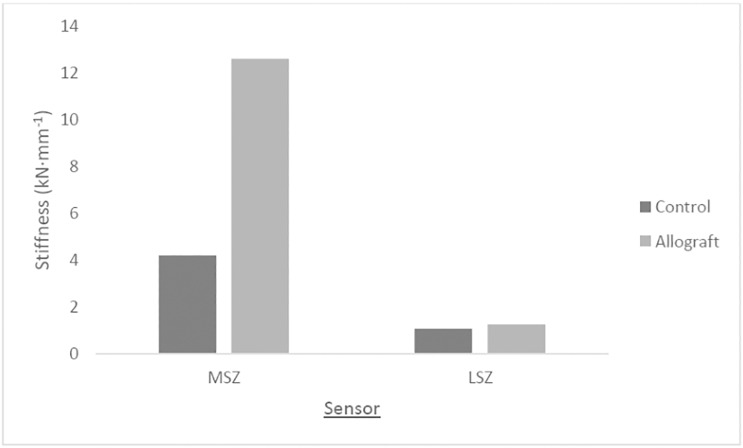
Mean specimen static stiffness around the tibial head at the point of failure.

Fig 8 shows the mean stiffness for each group at each sensor position around the tibial head. The allograft group exhibited higher specimen stiffness than the control group. The largest difference in stiffness between groups was seen at position MSZ. The lateral side of the tibial head showed the lowest overall stiffness in both groups compared to the medial side.

Table 2 gives the p-values obtained after comparing the mean values of the peak force and of the different stiffnesses in each group. All the p-values were >0.05. Therefore, the differences were statistically non-significant.

**Table 2:** p-values obtained after the t-test.

	Peak Force	Stiffness at time of break					
		LSX	MSY	LSY	MSZ	LSZ	VS
p-value	0.31	0.46	0.79	0.17	0.46	0.46	0.67

### Fatigue strength tests

Table 3 shows the load step, the approximate number of cycles, and maximum sinusoidal force that was being applied to each specimen at the point of failure. Specimen “control 1” performed best, reaching the highest load step, and therefore withstanding more cycles and higher forces, than all other specimens. The remaining specimens from the control group, performed inferiorly to those in the allograft group.

**Table 3:** Load step, approximate number of cycles, and maximum sinusoidal force at time of specimen failure.

Specimen	Load Step in which Fracture Occurred	Approximate Number of Cycles Until Failure	Maximum Sinusoidal Force (kN)
Control 1	4	67, 308	1.12
Control 2	2	37,974	0.80
Control 3	2	20,037	0.80
Allograft 1	3	42,630	0.96
Allograft 2	2	39,341	0.80

The vertical (VS) and lateral (LSZ) dynamic stiffness of each specimen undergoing fatigue strength testing was analysed, following the protocol of Diffo Kaze *et al*. (2015). A trend towards the lateral side of the tibial head being stiffer than the overall vertical dynamic stiffness could be seen in the control group, whereas the opposite was true for the allograft group. Specimen 3 in the control group exhibited weaker lateral dynamic stiffness in comparison to the other control specimens. The difference between the mean values of the control group and the allograft group was statistically non-significant (p >0.05).

## Discussion

The results of this study show that inserting an allograft during MOWHTO with large (>10°) corrections gives superior support and strength to the construct compared with osteotomies where no graft is used. During static compression, both groups fractured under a force greater than the physiological knee loads during normal, level walking (about 3 times bodyweight) [22]. The allograft group withstood higher forces than the control group prior to construct failure, which may be explained by the added medial and lateral stiffness of the tibial head provided by the wedge (Fig 8). This added static stiffness may have reduced valgus malrotation of the tibial head, which likely helped to distribute the vertical force more evenly across the tibial head and lowered the stress on the lateral cortex, the weakest point of a MOWHTO [5, 21, 23]. Furthermore, a recent study [24] used a 3D finite element model to find that the way that loads are balanced between the medial and lateral compartments of the knee may be key in optimising the clinical outcome of the procedure. The added stiffness that the allograft wedges provided the osteotomy construct in our study, in particular to the lateral cortex, may indicate that their inclusion could be a method of better distributing compressive and shear forces across the knee, leading to better outcomes clinically. This would be particularly relevant for larger correction angles, which have been previously associated with inferior outcomes [12, 13, 16].

The largest difference in displacement between groups was at position MSZ, the medial side of the tibial head. This is also where the Tomofix plate was fixed, and where the allograft was at its thickest, explaining the large discrepancy within groups between the medial and lateral sides of the tibial head. With the exceptions of LSY and MSZ, larger absolute displacements were seen in the allograft group. This would be expected due to the displacement controlled nature of the test protocol (with the piston moving at a constant rate of 0.1 mm·s^-1^), meaning longer tests will result in larger displacements than in specimens that fail at lower loads. However, the fact that displacements were observed in the x, y, and z-axes of the transverse plane, suggests that the tibial head moves and rotates in multiple directions as forces are applied to it. Therefore, it can be inferred that providing as much stability as possible to the construct is of vital importance in the earlier stages of healing, particularly given that more evidence is emerging that advocates the use of early weight bearing for knee osteotomy patients [25–29].

If it is assumed that a person moving without restriction will perform approximately 1 million cycles of the knee in a year [30], the specimens in the present study survived the equivalent of around 2 weeks (allograft group) and 1–4 weeks (control group) before failure. Given that it takes approximately 2 weeks for soft callus formation to begin to occur [20], the fatigue tests demonstrated the importance of restricting the forces applied to a large osteotomy where no healing has taken place, due to the high likelihood of construct failure. It must be remembered that the present study was conducted *in-vitro* and that these results only approximate *in-vivo* efficacy since full, unrestricted, weight bearing of the knee would only occur at least 11 days after surgery in patients specifically undergoing an early weight bearing rehabilitation protocol [3, 25, 28, 31]. Moreover, in cases where there is an intraoperative lateral hinge fracture, as with the specimens in the present study, weight bearing post-surgery may be delayed to allow some healing to take place [29].

The incidence of intraoperative lateral hinge fractures in the present study aligns with the findings of previous studies stating that such complications are particularly likely to occur in openings of >8° [21]. Intraoperative hinge fractures also negatively influence construct stability [6], causing a higher rate of correction loss and non-union to occur in such cases [12, 32]. This perhaps suggests that maximising construct stability in large corrections, or in cases with hinge fractures, is advisable not only for biomechanical reasons but also from a clinical perspective.

The allograft group exhibited the highest stiffness across the tibial head while under static compression. The largest difference in static stiffness between groups was seen at MSZ, the medial side of the osteotomy where the graft was at its thickest. This could be interpreted as further support to the conclusion that allografts provide additional stability to the construct, even at the point that is the strongest [5]: the medial side where fixator plate is located.

Despite the abovementioned findings from the specimens that underwent static compression, the differences between groups after fatigue strength testing are subtler. There does not appear to be any significant difference between groups in the data displayed in Table 1, however it does seem that there are far more variations in performance between specimens in the control group than within the allograft group.

The dynamic stiffness figures of the specimens, which underwent fatigue strength testing, show that lateral dynamic stiffness seems generally to be similar between groups, but that vertical dynamic stiffness appears to be slightly increased in the allograft group. This provides further evidence that the graft provides additional stability to the construct as a whole, but that the volume of the graft is important, and that at the point at which the graft is at its thinnest–the lateral cortex of the tibial head–less support is yielded.

A disturbance was at ~4000 seconds in the vertical dynamic stiffness of the allograft group, but not in the control group. 4000 seconds is the point at which the second load step began. The disturbance at this point suggests that the graft was resisting to the increase in the maximum force being applied to it. Specimen 1 from the Allograft Group also displayed a large and sudden increase in dynamic stiffness at approximately 6500 seconds, before returning to previous levels. This may indicate that the graft was cracking or breaking. This is further supported by the fact that this phenomenon occurred towards the end of the test.

The findings in present study are limited by the small sample size, and, as such, further research into this area is recommended. Furthermore, since the testing was conducted *in-vitro* with vertical force being applied perpendicular to the tibial plateau, the multi-axial forces that would be applied by the surrounding soft tissue in an *in-vivo* study were not considered.

Artificial bones were used in the present study in order to standardise the specimens and reduce the variability that has led to large differences in published results from cadaveric studies [18]. Although the bones used in the present study were artificial, they have been shown to approximate the biomechanical properties of human bone [18, 19]. However, further biomechanical analyses into the inclusion of bone grafts in MOWHTO using cadaveric specimens could be useful. As a result of this and the aforementioned limitations, all conclusions drawn from the present study should only be used as a general indication of allograft performance in MOWHTO and caution should be exercised when seeking to apply these findings to a clinical setting.

## Conclusion

Medial opening wedge high tibial osteotomy with allograft augmentation is a more stable construct than without a graft. This finding may be of significant importance in patients requiring a large correction, or in cases of lateral hinge fracture. Valgus malrotation of the tibial head is reduced when an allsograft is inserted into the osteotomy gap, which may help to protect the lateral cortex post-operatively.

Superior and more consistent biomechanical properties have been observed in MOWHTO with allograft augmentation, which could lead to more predictable outcomes in clinical settings.
